# Novel Protein Kinase Inhibitors Related to Tau Pathology Modulate Tau Protein-Self Interaction Using a Luciferase Complementation Assay

**DOI:** 10.3390/molecules23092335

**Published:** 2018-09-12

**Authors:** Max Holzer, Nico Schade, Ansgar Opitz, Isabel Hilbrich, Jens Stieler, Tim Vogel, Valentina Neukel, Moritz Oberstadt, Frank Totzke, Christoph Schächtele, Wolfgang Sippl, Andreas Hilgeroth

**Affiliations:** 1 Department for Molecular and Cellular Mechanisms of Neurodegeneration, Paul Flechsig Institute for Brain Research, University of Leipzig, Liebigstraße 19, 04103 Leipzig, Germany; max.holzer@medizin.uni-leipzig.de (M.H.); isabel.hilbrich@medizin.uni-leipzig.de (I.H.); jens.stieler@medizin.uni-leipzig.de (J.S.); tim.vogel.le@gmail.com (T.V.); valentina.neukel@hs-furtwangen.de (V.N.); 2 Institute of Pharmacy, Martin-Luther-University Halle-Wittenberg, 06120 Halle, Germany; nico.schade@pharmazie.uni-halle.de (N.S.); ansgar.opitz@pharmazie.uni-halle.de (A.O.); wolfgang.sippl@pharmazie.uni-halle.de (W.S.); 3 Department of Neurology, University of Leipzig, Liebigstraße 20, 04103 Leipzig, Germany; moritz.oberstadt@medizin.uni-leipzig.de; 4 ProQinase GmbH Freiburg, Breisacher Straße 117, 79106 Freiburg, Germany; f.totzke@proqinase.com (F.T.); c.schaechtele@proqinase.com (C.S.)

**Keywords:** AD drug discovery, synthesis, derivatives, structure-activity, lead structure

## Abstract

The current number of drugs available for the treatment of Alzheimer’s disease (AD) is strongly limited and their benefit for therapy is given only in the early state of the disease. An effective therapy should affect those processes which mainly contribute to the neuronal decay. There have been many approaches for a reduction of toxic Aβ peptides which mostly failed to halt cognitive deterioration in patients. The formation of neurofibrillary tangles (NFT) and its precursor tau oligomers have been suggested as main cause of neuronal degeneration because of a direct correlation of their density to the degree of dementia. Reducing of tau aggregation may be a viable approach for the treatment of AD. NFT consist of hyperphosphorylated tau protein and tau hyperphosphorylation reduces microtubule binding. Several protein kinases are discussed to be involved in tau hyperphosphorylation. We developed novel inhibitors of three protein kinases (gsk-3β, cdk5, and cdk1) and discussed their activity in relation to tau phosphorylation and on tau–tau interaction as a nucleation stage of a tau aggregation in cells. Strongest effects were observed for those inhibitors with effects on all the three kinases with emphasis on gsk-3β in nanomolar ranges.

## 1. Introduction

Alzheimer’s disease (AD) is a worldwide disease affecting more and more people preferably in the higher years of age [1,2]. It is predicted that by 2050 one of 85 people globally will have AD [3,4]. The progressive neurodegenerative disease is characterized by deteriorating cognitive functions and a loss of memory [1,5]. Presently, the disease cannot be cured even if it is recognized at an early state. The clinically available therapeutics are acetylcholine esterase inhibitors that increase the neuronal availability of the lowered acetylcholine levels [1,6,7]. Memantine is used as *N*-methyl-d-aspartate glutamate receptor antagonist to prevent an ongoing stimulation of excitatoric neurons [8]. However, those drugs cannot combat the cause of the disease. Thus, the drugs’ benefit is limited to just the early stage of disease.

Aβ peptides processed from the amyloid precursor protein (APP) by the activity of β- and γ-secretase form neurotoxic oligomers by aggregation [1,6]. Secretase inhibitors were partly disappointing in clinical trials so far with an observed worsening of cognitive functions [6,9,10,11]. Other Aβ-related strategies to prevent amyloid aggregation or remove amyloid by immunotherapeutics failed so far to halt cognitive deterioration [6,9,12]. Reasons for that have been critical side effects or a doubtful efficiency for a perspective clinical outcome [6,13].

Another key element in AD is hyperphosphorylated tau protein that forms tau filaments which further assemble to aggregates named neurophil threads and neurofibrillary tangles [6,14,15]. The density of those aggregates is well correlated with the degree of dementia in most studies of AD patients [16]. Therefore, a reasonable strategy has been to identify effective tau aggregation blockers. Although first experimental results have been promising, no compounds progressed into clinical practice so far [1,17,18].

Another strategy may be the prevention of tau hyperphosphorylation by the inhibition of tau kinases. Tau protein with an abnormal high degree of phosphorylation is hindered from binding to microtubules increasing free tau protein concentration and is missorted to the somatodendritic compartment. A major tau kinase is the glycogen synthase kinase-3β (gsk-3β) [19]. Early inhibitors such as indirubins [20], maleimides [21] and thiazoles [22] of gsk-3β have been ATP-competitive, but did not reach clinical trials due to a limited specificity and toxic effects [23]. Lithium as almost weak and noncompetitive inhibitor showed promising effects in used mouse models of tauopathies to prevent the development of tau pathology in the early stage of the disease [24]. From other noncompetitive inhibitors such as the thiadiazolidindiones (TDZDs) and the alkaloid manzamine with an allosteric binding mode tideglusip as TDZD reached clinical trials with a so far disappointing efficiency [25,26,27]. Alternatively, substrate-competitive inhibitors with a peptide scaffold have been developed which are phosphorylated by gsk-3β and then react as inhibitors [25,28]. In an AD mouse model, they showed a lowered Aβ load with reduced Aβ aggregates and fibrils detected [28]. Irreversible gsk-3β inhibitors of the halomethylketones presently showed only weak inhibitory activity [25]. One published multitarget inhibitor of gsk-3β of the 2,4-dihydropyrano[2,3-*c*]pyrazole family showed inducing in vitro effects on antioxidant and anti-inflammatory enzymes which result from the inhibition of gsk-3β at micromolar concentrations [29].

We wondered whether a kinase inhibitor with a broader activity towards tau-relevant kinases such as cdk5 and cdk1 may be a promising alternative for a perspective AD drug development. In addition, we tested whether such inhibitors not only affect tau phosphorylation but as a functional read-out also attenuate tau protein self-interaction because tau-dimer formation is the rate-limiting step in tau aggregation [30].

We developed novel small-molecule inhibitors with a benzofuropyridine scaffold and evaluated their properties to inhibit three most prominent kinases related to tau pathology. One earlier benzofuropyridine showed some cdk1 inhibitory properties beside a residual gsk-3β affinity [31]. Compound structure-dependent effects on kinase inhibition are discussed and first lead compounds could be identified. A preliminary proof-of-concept for a perspective reduced tau aggregation was evaluated in a tau interaction assay system to reflect a correlation of the compounds potency of kinase inhibition and a reduced tau interaction. Here, the roles of both the number and the single kinase potency are discussed related to the assay aggregation results.

## 2. Results and Discussion

### 2.1. Synthesis of the Benzofuropyridines

**Scheme 1:**
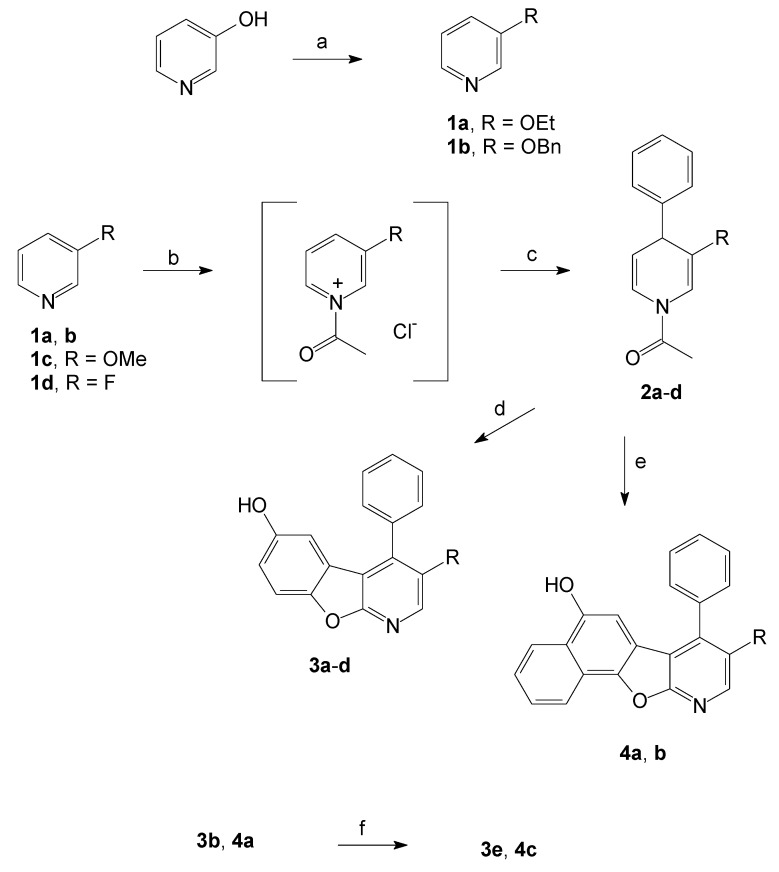
Synthesis of the benzofuropyridines **3a**–**e** and **4a**–**c**: (**a**) KOH, nBu_4_NBr, alkyl Br, THF, 12 h reflux; (**b**) CuI, Me_2_S, MeCOCl, THF, 15 min −20 °C; (**c**) PhMgCl, THF, 15 min −20 °C; (**d**) *p*-benzoquinone, HClO_4_, dioxane; (**e**) 1,4-naphthoquinone, HClO_4_, dioxane; and (**f**) H_2_, Pd/C, MeOH, 2 bar.

First, the 3-alkoxy pyridines **1a** and **b** were synthesized from 3-hydroxypyridine by the use of the corresponding alkyl bromides in a strong alkaline THF solution and tetra-*n*-butyl ammonium bromide as phase transfer catalyst (Scheme 1).

The pyridine compounds **1a**–**d** were then dissolved in THF under copper(I) iodide and dimethyl sulphide addition. After cooling down the temperature to −20 °C, acetyl chloride was added dropwise and the reaction mixture was stirred for 15 min at the low temperature. Then, phenyl magnesium chloride was added as solution in THF and stirring continued for additional 15 min. After work up of the mixture, the 1,4-dihydropyridines **2a**–**d** were yielded from a methanol solution under cooling. 

A double set of proton signals was found in the compound spectra due to the existence of two sets of isomers A and B, one with the acetyl function on the right site of the molecule as isomer A and one with that acetyl function on the left side of molecule as isomer B. The isomers result from a hindered rotability of the C-acetyl-N-pyridine bond due to a double bond character of that bond typical for such amide bonds. 

The 1,4-dihydropyridines were then dissolved in dry dioxane containing 10% perchloric acid and the corresponding quinones were added to give either the benzoquinone compounds **3a**–**d** or the naphthoquinone compounds **4a**,**b**. The reaction mechanism is suggested to start with a primary Michael addition of the electrophilic protonated quinone to the nucleophilic C-5 of the 1,4-dihydropyridine. Then, one quinone oxygen might have attacked the C-6 atom of the dihydropyridine to form a tetrahydro cycloaddition product. Excess of quinone then aromatized the scaffold under removing the acetyl residue to give the benzofuropyridine target compounds. Finally, the benzyl residues in the 3-benzyloxy compounds **3b** and **4a** were cleaved in methanol using hydrogen and palladium/charcoal as catalyst and a pressure of 2 bar to give the 3-hydroxy compounds **3e** and **4c**, respectively.

Spectroscopical target compound characteristics have been a low field shift of the 5-protons next to the hydroxyl function to about 6.5 ppm resulting from a shielding effect of the 4-phenyl residue that does not lie in a plane with the molecular scaffold due to the 3-substituents.

### 2.2. Protein Kinase Inhibition 

Tau protein hyperphosphorylation is mediated by an imbalance of protein kinases and phosphatase activity. Most prominent kinase has been gsk-3β that is discussed to interfere additionally with Aβ pathology [32]. A constitutive activation of the cyclin dependent kinase 5 (cdk5) by p25 as the truncated cdk5-regulative subunit p35 is discussed as causative for the cdk5 overactivation in AD brains, thus contributing to the tau hyperphosphorylation [33]. Moreover, recent studies prove that overactive cdk5 induces cell cycle regulating kinase cdk1 [34]. Such an aberrant cdk1 activation triggers neuronal death and thus potentiates the AD pathology. Thus, an inhibition of those kinases may have a strong benefit to prevent the tau-mediated AD pathology.

The protein kinase inhibitory activity has been determined in an assay system using the respective kinases, the kinase substrates and ATP with incorporated radioactive phosphorus P^33^ together with increasing inhibitor concentrations. The degree of the reduced substrate phosphorylation has been determined by scintillation counting and the inhibitory compound activity has been calculated accordingly. The results are shown in Table 1.

The 3-ethoxy function in compound **3a** resulted in a submicromolar affinity towards cdk1 and cdk5 and a nanomolar one towards gsk-3β. The replacement of the ethyl with a benzyl function in compound **3b** was less tolerated by all the kinases with decreases in activity and a best micromolar activity towards cdk1. The 3-methoxy function in compound **3c** resulted in similar affinities towards all the three kinases than the 3-ethoxy substitution. If the 3-alkoxy function was replaced with a hydroxyl function in derivative **3e** we found best activities towards all the kinases reaching nanomolar ranges for both cdk1 and gsk-3β and a lowest submicromolar affinity towards cdk5. A oxygen-free 3-fluoro function in compound **3d** was less favourable. However, we found better affinities towards gsk-3β in submicromolar and towards cdk1 in low micromolar ranges than those determined for the 3-benzyloxy substituted compound **3b**.

Next, we evaluated the benzo-annelated compounds **4a**–**c**. The benzo-annelated compound **4a** with the 3-benzyloxy substitution showed an overall improved kinase inhibitory activity compared to compound **3b** without the additional phenyl ring. The cdk1 inhibitory activity reached nanomolar ranges, the gsk-3β affinity increased and a micromolar cdk5 activity was found. The 3-fluoro compound **4b** with the additional phenyl ring showed a slightly improved cdk1 affinity compared to compound **3d** without the attached phenyl residue. The gsk-3β affinity was just residual similar to a determined cdk5 activity that was not found for compound **3d**. The attached phenyl residue in derivative **4c** with the 3-hydroxy function resulted in almost unchanged activities if compared to the derivative **3e** without that phenyl substituent. However, best nanomolar affinities towards cdk5 and gsk-3β were reached for compound **4c**.

Overall, it can be stated that the 3-hydroxy function in both compound types **3** and **4** resulted in best kinase inhibitory activities.

Docking studies have been carried out to demonstrate the binding mode and explain observed activities of respective compounds. The 3-hydroxy compounds **3e** and **4c** are able to form three hydrogen bridge bonds to amino acid residues of the protein backbone in the ATP binding pocket as demonstrated for compound **3e** in the case of gsk-3β with bonds of the 3-hydroxy function to asparagine200 via a localized water molecule, the 6-hydroxy function with a direct bond to the NH function of arginine141 and, finally, the pyridine nitrogen with a bond to the NH function of valine135 of the catalytic site (see Figure S1 in the Supplementary Materials). If the 3-hydroxy function is replaced with a benzyloxy residue only two hydrogen bridge bonds are observed for compound **4a** to explain the lowered activity. Docking to both cdk1 and cdk5 shows each hydrogen bridge bonds of the 6-hydroxy function of **4a** to the carbonyl function of isoleucine10 of each enzyme and the pyridine nitrogen to the respective amino acid of the catalytic site of cdk1 with leucine83 and of cdk5 with cysteine83 (see Figure S2a,b in the Supplementary Materials). The benzyloxy residue shows hydrophobic interactions with each phenyl residue of phenylalanine80 as gatekeeper amino acid. In compound **4b** with a fluoro substitution such interactions are not possible and thus a decrease in activity was observed.

A first protein kinase profiling has been carried out with compound **3e** which was evaluated to inhibit kinases of five kinase families. We found no activity towards kinases of the PKA family (PKC-α, -γ, -ε and -iota), towards kinases of the receptor tyrosine kinase family (VEGFR2, ErBB2 and TIE2), and towards WEE1 and CK1-α of the casein kinase family. Towards cdk6 of the family of the cyclin dependant kinases the activity was 829 µM and towards EGFR of the receptor tyrosine kinases 523 µM. Finally, the activity towards Src of the SRC family was evaluated with 230 µM.

**Figure 1:**
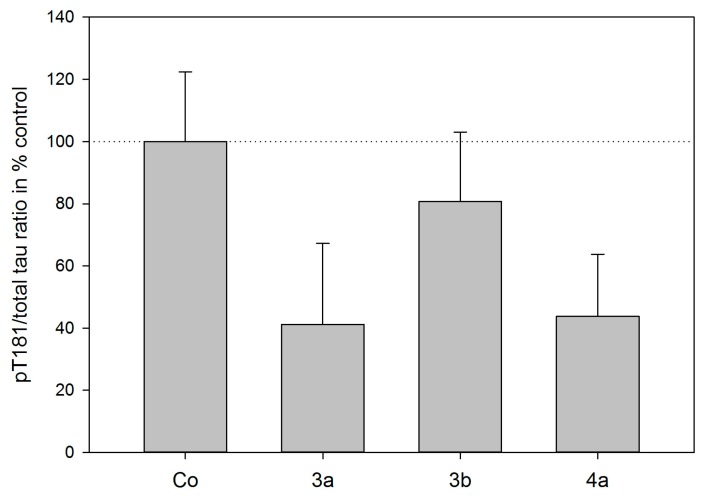
Quantification of threonine 231 und serine 235 tau phosphorylation after 24 h inhibitor treatment at 8 µM concentration of 2N4R-tau transfected COS-7 cells by Western blot analysis. Phospho-dependent immunoreactivity was normalized to total tau immunoreactivity and depicted as mean ± SD.

Next, we investigated three of our compounds **3b**, **4a** and **3a** to inhibit the tau phosphorylation in tau transfected COS-7 cells. They showed increasing activities towards the kinases from **3b** to **3a** as discussed and thus were expected to result in a compound-dependent reduced tau phosphorylation related to a lowered activity. Moreover, they were selected with a favourite solubility in the assay system. We used AT180 monoclonal antibody to detect the tau phosphorylation of threonine 231 and serine 235 as relevant amino acids for tau phosphorylation by the respective kinases using Western blot technique. The results are shown in Figure 1.

Compound **3b** with a just micromolar affinity towards two kinases (cdk1, gsk-3β) resulted in a reduction of amino acid phosphorylation of 19% compared to the untreated control. Compound **4a** with an additional nanomolar affinity towards cdk1 reduced tau amino acid phosphorylation by 55%. Finally, compound **3a** with an increased submicromolar affinity towards two kinases (cdk5, gsk-3β) reduced the tau phosphorylation by 61%. The data demonstrate an expected correlation of the degree of protein kinase inhibition with the tau phosphorylation in living cells of AD-relevant phosphorylation.

### 2.3. Tau Protein Interaction Assay

An overall increase in tau phosphorylation results in a reduced microtubule binding and increased pool of free tau protein, which is more prone to aggregation by laws of mass action [35]. Protein complementation assays have recently been employed to visualize and measure protein oligomerization and aggregate propagation of amyloidogenic proteins such as tau protein, SOD1, TDP43 or alpha synuclein in cultured cells [36,37,38,39,40].

In our tau protein interaction assay in living cells, the intracellular luciferase activity is reconstituted, when two or more tau molecules oligomerize and come in close contact, thereby aligning the two split luciferase fragment in close proximity. Two different NanoLuc luciferase subunits (large Bit (lgBit), an 18 kDa polypeptide, and small bit (smBit), a 1.3 kDa peptide) have been fused to the N-terminal and C-terminal site of 441aa 2N4R human tau isoform, respectively. This optimal configuration (N-terminal or C-terminal fusion) has been tested. The NanoBit subunits only weakly associate by themselves, so that the reconstitution of luciferase activity is only dependent on tau protein self-interaction. A human liver cell line (HuH-7), devoid of endogenous tau protein was transfected with the two fusion constructs and after 24 h post-transfection cells were subcultured in a 384-well plate for inhibitor treatment and luminescence detection. Further, to detect off-target effects of compounds such as toxicity or interference with luciferase reaction, protein homeostasis and thymidine kinase promoter activity, we applied the same compounds onto HuH-7 cells transfected with a construct expressing full length constitutively-active NanoLuc luciferase driven by the same promoter like the tau-fusion constructs. Therefore, results of tau protein self-interaction were normalized with respect to full length NanoLuc activity after inhibitor treatment to even rule out such side effects. Toxicity at 10 µM inhibitor concentration has additionally been measured using the WST-8 assay as described [39] and has been found negligible.

The assay is a straightforward method to determine the effects on tau protein self-interaction in a short time-frame of 60 min, where effects are mainly caused by direct effects on tau phosphorylation or indirect by affecting tau scaffolding proteins and can be related to the kinase inhibitors potency. The luminescence signal determined for vehicle control (0.1% DMSO) is set to 100% and increase or decrease of interaction is given in percent of vehicle control.

Thus, an observed correlation of the protein kinase inhibitory potential of our compounds with the reduction of tau self-interaction would be a proof-of-concept that lesser phosphorylated tau regained microtubule binding and/or has a reduced propensity for self-interaction, which reduces the pool of free tau protein prone to tau aggregation.

**Figure 2:**
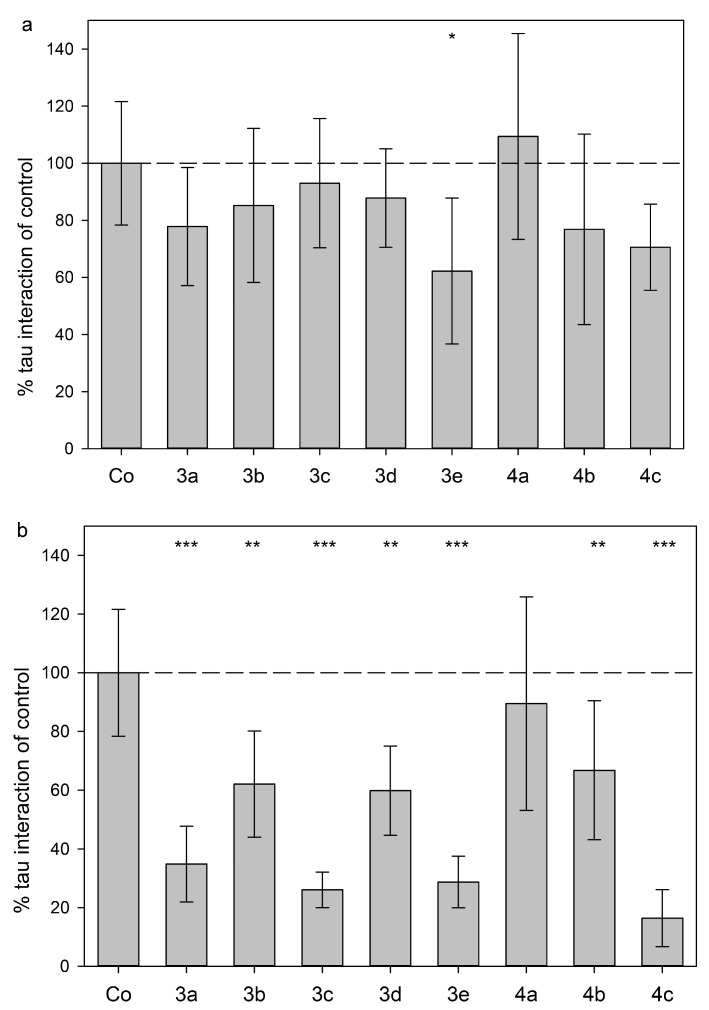
Modulation of tau interaction by protein kinase inhibitors. Tau protein self-interaction was assessed using a split-luciferase assay. Luminescence readings were related to vehicle control (Co) and normalized to the full length constitutively-active NanoLuc luciferase and depicted as mean ± SD. (**a**) Inhibitor concentration of 1 µM and (**b**) inhibitor concentration of 10 µM. * *p* < 0.05; ** *p* < 0.01, *** *p* < 0.001.

The two 3-hydroxy substituted compounds **3e** and **4c** with nanomolar protein kinase inhibitory activities towards two kinases and highest gsk-3β inhibitor potency showed a reduction of luminescence of 38% and 29% at the lower concentration of 1 µM (Figure 2a, above), which resulted in a higher inhibition of 71% and 84% at the 10 µM inhibitor concentration, respectively (Figure 2b).

Both 3-alkoxy substituted compounds **3a** and **3c** caused only a mild inhibition of tau interaction at a 1 µM concentration of 7% for the 3-methoxy substituted derivative **3c** and of 22% for the 3-ethoxy substituted of compound **3a**. Tau interaction is inhibited by 65% (**3a**) and 75% (**3c**) at the higher concentration of 10 µM that may be related to a partly reduced protein kinase inhibitory activity in nanomolar ranges towards just one kinase compared to best compounds **3e** and **4c**. The 3-benzyloxy substitution of compound **3b** with just micromolar affinities towards two kinases resulted in a weak inhibitory activity of 15% at 1 µM and with a just 38% reduced luminescence at 10 µM, similar to the 3-fluoro substitution of compound **3d** with a 12% luminescence reduction at the lower and 40% at the higher inhibitor concentration and a reduced compound activity towards one kinase in submicromolar and one in micromolar ranges. The benzo-annelated compound **4b** at a 1 µM concentration caused a luminescence inhibition of 23% compared to a 12% inhibition of compound **3d** without that phenyl substitution. Using the benzo-annelated compound **4c**, we found an increased luminescence inhibition compared to compound **3e** at 10 µM.

Summarizing our results, it can be stated that the split luciferase interaction assay correlates with the differential protein kinase inhibitory activity of the compounds. Kinase inhibitors with a nanomolar *K*_i_ towards gsk-3β in combination with a nanomolar inhibition of a second kinase cdk5 or cdk1 proved the best inhibitors of tau self-interaction. Only compound **4a** showed an unexpected low influence in the tau interaction that may be plausible with a partial observed precipitation of the compound in the assay system. Cellular compound toxicity studies using the WST-8 assay [39] have been carried out to demonstrate a non-toxicity of the compounds, so that a reduced luminescence under compound application is not influenced by a cellular compound toxicity (data not shown). As proof-of-concept it can be stated that a kinase inhibitor-induced reduced tau phosphorylation leads to a lowered tau interaction and may diminish tau pathology by influencing tau aggregation.

## 3. Material and Methods

### 3.1. Chemical Reagents and Instruments

Commercial reagents were used without further purification. The ^1^H-NMR spectra (400 MHz) were measured using tetramethylsilane as internal standard. TLC was performed on E. Merck 5554 silica gel plates. The high-resolution mass spectra were recorded on a Finnigan LCQ Classic mass spectrometer. Elemental analysis indicated by the symbols of the elements was within ±0.4% of the theoretical values and was performed using a Leco CHNS-932 apparatus.

### 3.2. General Procedure for the Synthesis of Compounds ***1***

Five grams (52.6 mmol) 3-hydroxypyridine, 5.9 g (110 mmol) pulverized potassium hydroxide and 0.85 g (2.6 mmol) nBu_4_NBr were dissolved in 150 mL of dried THF. After addition of 84 mmol of the respective alkyl halogenide the mixture was stirred for 12 h under reflux. The reaction was finished with the addition of 200 mL water and extraction with each 100 mL of hydrochloric acid (10%) for two times. The unified water phases were alkalined with a 10 M sodium hydroxide solution and then extracted with each 75 mL of chloroform for three times. After removal of the solvent of the unified organic layers in vacuum, the raw product was purified using column chromatography technique over silica gel and an eluent mixture of cyclohexane and ethyl acetate in a relation of 1:1.

*3-Ethoxypyridine* (**1a**). Yield 78%; IR (ATR) *ν* = 1260 (C-O-C) cm^−1^; ^1^H-NMR (CDCl_3_) δ = 8.26 (dd, *J* = 2.7, 0.8 Hz, 1H, 2-H), 8.16 (dd, *J* = 4.4, 1.7 Hz, 1H, 6-H), 7.16 (ddd, *J* = 8.4, 4.2, 0.8 Hz, 5-H), 7.12 (ddd, *J* = 8.4, 2.7, 1.7 Hz, 1H, 4-H), 4.03 (q, *J* = 7 Hz, 2H, OC**H**_2_CH_3_), 1.39 (t, *J* = 7 Hz, 3H, OCH_2_CH_3_); *m*/*z* (ESI) 124.16 (M + H^+^).

*3-Benzyloxypyridine* (**1b**). Yield 59%; IR (ATR) *ν* = 1260 (C-O-C) cm^−1^; ^1^H-NMR (acetone-*d_6_*) δ = 8.37 (d, *J* = 2.9, Hz, 1H, 2-H), 8.18 (dd, *J* = 4.6, 1.3 Hz, 1H, 6-H), 7.57–7.5 (m, 2H, 2′-, 6′-H), 7.41–7.31 (m, 4H, 4-H, 3′-, 4′-, 5′-H), 7.26 (ddd, *J* = 8.4, 4.6, 0.6 Hz, 1H, 5-H), 5.18 (s, 2H, OC**H**_2_C_6_H_5_); *m*/*z* (ESI) 186.28 (M + H^+^).

### 3.3. General Procedure for the Synthesis of Compounds ***2***

First, 10 mmol of the respective pyridine were dissolved in 50 mL of dried THF. After addition of 0.5 mmol of copper(I) iodide and 3 mL of dimethyl sulphide, the mixture was stirred at room temperature until a clear solution resulted. Then, it was cooled down to −20 °C. After 15 min, 10 mmol of acetyl chloride were added dropwise and stirring continued for 15 min. Then, 10 mmol of phenyl magnesium chloride were added and stirring followed for additional 15 min. Then, the mixture warmed up to room temperature. Then, 50 mL of an ammonium chloride solution (20%) were added and extraction with diethyl ether followed with portions of 50, 25 and again 25 mL. The unified organic layer was extracted with each 50 mL of a solution of ammonium chloride in ammonia for two times, with 50 mL water, hydrochloric acid (10%) for two times, water and finally a saturated solution of sodium chloride in water. The organic layer was then dried over sodium sulphate, filtered and finally removed under reduced pressure to give an oily substance that partially crystallized from methanol.

*1-(3-Ethoxy-4-phenyl-4H-pyridine-1-yl)ethanone* (**2a**). Yield 56%; mp 124–129 °C: IR (ATR) *ν* = 1669 (**CO**CH_3_), 1633 cm^−1^; ^1^H-NMR (CDCl_3_) δ = 7.35–7.28 (m, 5H, 2′-, 6′-H of isomer A and B, 6-H of isomer A), 7.23–7.19 (m, 6H, 3′-, 4′-, 5′-H of isomer A and B), 6.86 (d, *J* = 1,2 Hz, 1H, 2-H of isomer B), 6.64 (dt, *J* = 8.2, 1.2 Hz, 1H, 6-H of isomer B), 6.06 (d, *J* = 1.2 Hz, 1H, 2-H of isomer A), 5.13 (dd, *J* = 8.2, 4.4 Hz, 1H, 5-H of isomer A), 5.04 (dd, *J* = 8.2, 4.4 Hz, 1H, 5-H of isomer B), 4.21 (d, *J* = 4.4 Hz, 2H, 4-H of isomer A and B), 3.74 (**AB**X_3_, *J* = 9.4, 7.0 Hz, 2H, OC**H**_2_CH_3_ of isomer B), 3.68 (**AB**X_3_, *J* = 9.0, 6.9 Hz, 2H, OC**H**_2_CH_3_ of isomer A), 2.25 (s, 3H, COCH_3_ of isomer B), 2.23 (s, 3H, COC**H**_3_ of isomer A), 1.20 (AB**X**_3_, *J* = 6.9 Hz, 3H, OCH_2_C**H**_3_ of isomer A), 1.17 (AB**X**_3_, *J* = 7.0 Hz, 3H, OCH_2_C**H**_3_ of isomer B); *m*/*z* (ESI) 244.31 (M + H^+^).

*1-(3-Benzyloxy-4-phenyl-4H-pyridine-1-yl)ethanone* (**2b**). Yield 58%; mp 78–82 °C: IR (ATR) *ν* = 1672 (**CO**CH_3_), 1635 cm^−1^; ^1^H-NMR (CDCl_3_) δ = 7.33–7.20 (m, 17H, OCH_2_C_6_**H**_5_ of isomer A and B, 3′-, 4′-, 5′-H of isomer A and B, 6-H of isomer A), 7.13–7.10 (m, 4H, 2′-, 6′-H of isomer A and B), 6.98 (d, *J* = 1,3 Hz, 1H, 2-H of isomer B), 6.67 (dt, *J* = 8.2, 1.3 Hz, 1H, 6-H of isomer B), 6.10 (d, *J* = 1.3 Hz, 1H, 2-H of isomer A), 5.15 (dd, *J* = 8.2, 4.3 Hz, 1H, 5-H of isomer A), 5.07 (dd, *J* = 8.2, 4.3 Hz, 1H, 5-H of isomer B), 4.77 (AB, *J* = 11.8 Hz, 2H, OC**H**_2_C_6_H_5_ of isomer B), 4.74 (AB, *J* = 11.9 Hz, 2H, OC**H**_2_C_6_H_5_ of isomer A), 4.31 (d br, *J* = 4.3 Hz, 2H, 4-H of isomer A and B), 2.26 (s, 3H, COCH_3_ of isomer B), 2.16 (s, 3H, COCH_3_ of isomer A); *m*/*z* (ESI) 306.38 (M + H^+^).

*1-(3-Methoxy-4-phenyl-4H-pyridine-1-yl)ethanone* (**2c**). Yield 40%; mp 50–70 °C: IR (ATR) *ν* = 1674 (**CO**CH_3_), 1633 cm^−1^; ^1^H-NMR (CDCl_3_) δ = 7.34 (dt, *J* = 8.2, 1.3 Hz, 1H, 6-H of isomer A), 7.34–7.28 (m, 4H, 2′-, 6′-H of isomer A and B), 7.24–7.20 (m, 6H, 3′-, 4′-, 5′-H of isomer A and B), 6.90 (d, *J* = 1,3 Hz, 1H, 2-H of isomer B), 6.64 (dt, *J* = 8.2, 1.3 Hz, 1H, 6-H of isomer B), 6.07 (d, *J* = 1.3 Hz, 1H, 2-H of isomer A), 5.12 (dd, *J* = 8.2, 4.3 Hz, 1H, 5-H of isomer A), 5.03 (dd, *J* = 8.2, 4.3 Hz, 1H, 5-H of isomer B), 4.23 (dd, *J* = 4.3, 1.3 Hz, 2H, 4-H of isomer A and B), 3.55 (s, 3H, OCH_3_ of isomer B), 3.52 (s, 3H, OCH_3_ of isomer A), 2.27 (s spl, 6H, COCH_3_ of isomer A and B); *m*/*z* (ESI) 230.28 (M + H^+^).

*1-(3-Fluoro-4-phenyl-4H-pyridine-1-yl)ethanone* (**2d**). Yield 61%; mp 69–70 °C: IR (KBr) *ν* = 1674 (**CO**CH_3_), 1638 cm^−1^; ^1^H-NMR (CDCl_3_) δ = 7.32 (dd, *J* = 2.7, 1.2 Hz, 1H, 2-H of isomer B), 7.35 (dd, *J* = 2.7, 1.2 Hz, 1H, 2-H of isomer A), 7.34–7.22 (m, 10H, C_6_**H**_5_ of isomer A and B), 6.69 (dd, *J* = 8.2, 1,6 Hz, 1H, 6-H of isomer A), 6.62 (dd, *J* = 8.2, 1.6 Hz, 1H, 6-H of isomer B), 5.18–5.11 (m, 1H, 5-H of isomer A), 5.09–5.02 (m, 1H, 5-H of isomer B), 4.44 (s br, 2H, 4-H of isomer A and B), 2.25 (s, 3H, COCH_3_ of isomer B), 2.21 (s, 3H, COCH_3_ of isomer A); *m*/*z* (ESI) 218.25 (M + H^+^).

### 3.4. General Procedure for the Synthesis of Compounds ***3*** and ***4***

First, 3.3 mmol of the respective compound **2** was dissolved in a minimum volume of freshly distilled dried dioxane. After addition of 4.1 mmol of the respective quinone, either 1,4-benzoquinone or 1,4-naphthoquinone, a mixture of dioxane and perchloric acid (70%) in a ratio of 22:1 (*v*:*v*) was added until a final solution volume of 75 mL was reached. The reaction proceeding was followed by thin layer chromatography (tlc). After 48 h of reaction 1.4 mmol of the quinone was added until no more starting or intermediate compounds were detectable by tlc. Then, 30 mL of water were added and the pH of the solution was adjusted to 8–9. Then, extraction with chloroform followed for three times using each half of the volume of the water phase. The unified organic layer was dried over sodium sulphate and filtered. The oily residue was purified by column chromatography over silica gel using an eluent mixture of cyclohexane and ethyl acetate in a ratio of 4:1 (*v*:*v*). After evaporation of the collected compound fractions, the target compounds crystallized from diethyl ether.

*3-Ethoxy-4-phenylbenzo[4,5]furo[2,3-b]pyridine-6-ol* (**3a**). Yield 10%; mp 231–235 °C; IR (KBr) *ν* = 3435 (OH), 1277 (C-O-C) cm^−1^; ^1^H-NMR (DMDO-*d_6_*) δ = 9.33 (s, 1H, OH), 8.25 (s, 1H, 2-H), 7.59–7.51 (m, 5H, C_6_H_5_), 7.49 (d, *J* = 8.9 Hz, 1H, 8-H), 6.91 (dd, *J* = 8.9, 2.5 Hz, 1H, 7-H), 6.51 (d, *J* = 2.5 Hz, 1H, 5-H), 4.09 (q, *J* = 7.0 Hz, 2H, OC**H**_2_CH_3_), 1.18 (t, *J* = 7.0 Hz, 3H, OC**H**_2_CH_3_); *m*/*z* (ESI) 306.35 (M + H^+^). Anal. (C_19_H_15_NO_3_) Calc. C 74.74, H 4.95, N 4.59; Found C 74.99, H 4.81, N 4.70.

*3-Benzyloxy-4-phenylbenzo[4,5]furo[2,3-b]pyridine-6-ol* (**3b**). Yield 13%; mp 220–224 °C; IR (KBr) *ν* = 3401 (OH), 1276 (C-O-C) cm^−1^; ^1^H-NMR (DMDO-*d_6_*) δ = 9.34 (s, 1H, OH), 8.32 (s, 1H, 2-H), 7.60–7.54 (m, 5H, C4-C_6_H_5_), 7.49 (d, *J* = 8.9 Hz, 1H, 8-H), 7.31–7.26 (m, 5H, OCH_2_C_6_**H**_5_), 6.92 (dd, *J* = 8.9, 2.7 Hz, 1H, 7-H), 6.51 (d, *J* = 2.7 Hz, 1H, 5-H), 5.17 (s, 2H, OC**H**_2_C_6_H_5_); *m*/*z* (ESI) 368.42 (M + H^+^). Anal. (C_24_H_17_NO_3_) Calc. C 78.46, H 4.66, N 3.81; Found C 78.95, H 4.59, N 3.76.

*3-Methoxy-4-phenylbenzo[4,5]furo[2,3-b]pyridine-6-ol* (**3c**). Yield 8%; mp 240–245 °C; IR (KBr) *ν* = 3434 (OH), 1275 (C-O-C) cm^−1^; ^1^H-NMR (DMDO-*d_6_*) δ = 9.34 (s, 1H, OH), 8.25 (s, 1H, 2-H), 7.61–7.53 (m, 3H, 3′-, 4′-, 5′-H), 7.51-7.49 (m, 2H, 2′-, 6′-H), 7.49 (d, *J* = 8.8 Hz, 1H, 8-H), 6.91 (dd, *J* = 8.8, 2.5 Hz, 1H, 7-H), 6.47 (d, *J* = 2.5 Hz, 1H, 5-H), 3.84 (s, 3H, OCH_3_); *m*/*z* (ESI) 292.32 (M + H^+^). Anal. (C_18_H_15_NO_3_) Calc. C 74.22, H 4.50, N 4.81; Found C 73.89, H 4.45, N 4.95.

*3-Fluoro-4-phenylbenzo[4,5]furo[2,3-b]pyridine-6-ol* (**3d**). Yield 6%; mp 266–274 °C; IR (KBr) *ν* = 3162 (OH), 1185 (C-O-C) cm^−1^; ^1^H-NMR (DMDO-*d_6_*) δ = 9.48 (s, 1H, OH), 8.51 (d, *J* = 2.3 Hz, 1H, 2-H), 7.68–7.66 (m, 5H, C4-C_6_H_5_), 7.59 (d, *J* = 8.9 Hz, 1H, 8-H), 7.00 (dd, *J* = 8.9, 2.6 Hz, 1H, 7-H), 6.70 (d, *J* = 2.6 Hz, 1H, 5-H); *m*/*z* (ESI) 280.27 (M + H^+^). Anal. (C_17_H_10_FNO_2_) Calc. C 73.11, H 3.61, N 5.02; Found C 72.97, H 3.69, N 4.97.

*3-Benzyloxy-4-phenylnaphtho[1′,2′:4,5]furo[2,3-b]pyridine-6-ol* (**4a**). Yield 6%; mp 219–222 °C; IR (KBr) *ν* = 3176 (OH), 1261 (C-O-C) cm^−1^; ^1^H-NMR (DMDO-*d_6_*) δ = 10.10 (s, 1H, OH), 8.37 (s, 1H, 2-H), 8.32 (d, *J* = 8.2 Hz, 1H, 10-H), 8.23 (d, *J* = 8.4 Hz, 1H, 7-H), 7.72 (t, *J* = 7.5 Hz, 1H, 9-H), 7.66–7.57 (m, 6H, C4-C_6_H_5_, 8-H), 7.38–7.25 (m, 5H, OCH_2_C_6_**H**_5_), 6.64 (s, 1H, 5-H), 5.20 (s, 2H, OC**H**_2_C_6_H_5_); *m*/*z* (ESI) 418.47 (M + H^+^). Anal. (C_28_H_19_NO_3_) Calc. C 80.56, H 4.59, N 3.36; Found C 80.23, H 4.35, N 3.25.

*3-Fluoro-4-phenylnaphtho[1′,2′:4,5]furo[2,3-b]pyridine-6-ol* (**4b**). Yield 2%; mp 246–248 °C; IR (KBr) *ν* = 3140 (OH), 1178 (C-O-C) cm^−1^; ^1^H-NMR (DMDO-*d_6_*) δ = 9.63 (s, 1H, OH), 8.87–8.82 (m, 2H, 2-, 10-H), 8.80 (d, *J* = 8.4 Hz, 1H, 7-H), 8.23-8.08 (m, 7H, C4-C_6_H_5_, 8-, 9-H), 7.27 (s, 1H, 5-H); *m*/*z* (ESI) 330.32 (M + H^+^). Anal. (C_21_H_12_FNO_2_) Calc. C 76.59, H 3.67, N 4.25; Found C 76.25, H 3.42, N 4.15.

### 3.5. General Procedure for the Synthesis of 3-Hydroxy Compounds ***3e*** and ***4c***

One equivalent of the respective 3-benzyloxy compound **3b** or **4a** was dissolved in 20 mL of distilled dried methanol at room temperature and 0.3 equivalent of palladium on charcoal (10%) were added. The solution was shaken under hydrogen atmosphere with a pressure of 2 bar. After 2–3 h, the reaction was stopped and the solution filtered. After removal of the solvent in vacuum, the 3-hydroxy compounds crystallized from diethyl ether.

*4-Phenylbenzo[4,5]furo[2,3-b]pyridine-3,6-diol* (**3e**). Yield 91%; mp 214–217 °C; IR (KBr) *ν* = 3350 (OH), 1183 (C-O-C) cm^−1^; ^1^H-NMR (DMDO-*d_6_*) δ = 9.72 (s br, 1H, OH), 9.29 (s br, 1H, OH), 8.07 (s, 1H, 2-H), 7.61–7.51 (m, 5H, C4-C_6_**H**_5_), 7.46 (d, *J* = 8.9 Hz, 1H, 8-H), 6.89 (dd, *J* = 8.9, 2.6 Hz, 1H, 7-H), 6.54 (d, *J* = 2.6 Hz, 1H, 5-H); *m*/*z* (ESI) 278.25 (M + H^+^). Anal. (C_17_H_11_NO_3_) Calc. C 73.64, H 4.00, N 5.05; Found C 73.38, H 3.85, N 4.99.

*4-Phenylnaphtho[1′,2′:4,5]furo[2,3-b]pyridine-3,6-diol* (**4c**). Yield 91%; mp 252–255 °C; IR (KBr) *ν* = 3337 (OH), 1172 (C-O-C) cm^−1^; ^1^H-NMR (acetone-*d*_6_) δ = 9.04 (s, 1H, OH), 8.49 (s, 1H, OH), 8.36 (d, *J* = 8.3 Hz, 1H, 10-H), 8.33 (d, *J* = 8.3 Hz, 1H, 7-H), 8.16 (s, 1H, 2-H), 7.74–7.70 (m, 1H, 9-H), 7.67–7.57 (m, 6H, C4-C_6_H_5_, 8-H), 6.71 (s, 1H, 5-H); *m*/*z* (ESI) 328.39 (M + H^+^). Anal. (C_21_H_13_NO_3_) Calc. C 77.05, H 4.00, N 4.28; Found C 76.79, H 4.12, N 4.21.

### 3.6. Protein Kinase Assay

The protein kinases were all expressed in Sf9 insect cells as human recombinant GST fusion proteins and purified by affinity chromatography using GSH-agarose. The kinase purity was finally checked by SDS-PAGE/Coomassie staining.

The measuring of protein kinase activity was performed in 96-well FlashPlates^TM^ in a 50 µL reaction volume. The reaction mixture consisted of 20 µL of assay buffer solution, 5 µL of ATP solution in water, 5 µL of used test compound in a 10% DMSO solution and finally a premixture of each 10 µL of used substrate and enzyme solutions. The assay buffer solution contained 70 mM of HEPES-NAOH, each 3 mM of magnesium chloride and manganese(II) chloride, 3 µM of sodium orthovanadate, 1.2 mM of DTT, 50 µg/mL of PEG20000 and finally 15 µM of [γ-^33^P]-ATP making approximately 1.2 × 10^6^ cpm per well.

The final kinase concentrations have been 3.5 nM for cdk1/cycline B, 3.3 nM for cdk5/p25, and finally 13.1 nM for gsk-3β. The substrate was RBER-CHKtide using amounts of 2000 ng/50 µL in the case of cdk1/cycline B and 1000 ng/50 µL in the case of cdk5/p25 and gsk-3β.

### 3.7. Tau Protein Phosphorylation Assay

COS-7 cells transfected with pDSRed2-humanTau (2N4R isoform) and cultured in DMEM/5% fetal calf serum for 24 h in 12-well culture plates were incubated with the respective compounds each 8 µM or 0.1% DMSO as vehicle control for 12 h at 37 °C. Cells were scraped, centrifuged for 10 min at 4 °C with 300× *g*, washed with cooled PBS and centrifuged again. Proteins were extracted with 300 µL PBS, 1% Triton X-100 including protease and phosphatase inhibitors (cOmplete^TM^, PhosSTOP^TM^, Roche Life Science, (Germany, Penzberg). Protein content was calculated by the Pierce 660 nm protein assay (Thermo Scientific, Rockford, IL, USA). Ten micrograms of protein from each sample were run as duplicates on 8% SDS-PAGE and transferred to a PVDF membrane. Total tau protein was labelled with a polyclonal total tau antibody (1:4000, *v*:*v*, #A0024, Dako, Glostrup, Denmark) and phospho-tau (pThr231, pSer235) was labelled with the monoclonal antibody AT180 (1:500, *v*:*v*, MN1040, Thermo Scientific). Western blots were imaged after incubation with anti-rabbit HRP/anti-mouse HRP (1:10,000, *v*:*v* NA934, NA931, GE Healthcare, Little Chalfont, UK) using the DNR Chembis ECL station and immunoreactive bands were quantified by densitometry using Tina 2.09 software (Raytest, Straubenhardt, Germany). Phospho-tau immunoreactivity was normalized to total tau immunoreactivity.

### 3.8. Tau Protein Interaction Assay

#### Cell Culture and Transfection

Human liver cell line (HuH-7) was maintained in Dulbecco’s modified Eagle’s minimum essential medium (DMEM/F-12) containing 9% foetal bovine serum. Transfection of the plasmids pFN33_tau and pFC36_tau (human 441aa 2N4R tau isoform fused to split luciferase fragments LgBit and SmBit, respectively) or pNL1.1.TK[Nluc/TK] vector (full length constitutively-active NanoLuc luciferase, Promega, Mannheim, Germany) in HuH-7 cells was performed in 6-well plates using the Lipofectamine^®^ 2000 Transfection reagent (ThermoFisher Scientific, Schwerte, Germany) according to manufacturer’s recommendation. Briefly, 2.5 µg DNA in 250 µL Opti-MEM^TM^ have been mixed with 6.2 µL of Lipofectamine^®^ 2000 in 250 µL Opti-MEM^TM^ incubated for 20 min and added to the HuH-7 cells per well. After 24 h incubation period, Huh-7 cells were plated into 384-well plates (3000 cells per well, µclear/white #781093 greiner bio-one) for inhibitor treatment and luciferase assay.

#### NanoBiT^®^ Luciferase Assay

The assay is based on complementation of a split luciferase nanoluc by the interaction of the tested proteins. For evaluation of the best configuration of the split-luciferase and tau fusion proteins, N-terminal and C-terminal fusion have been tested. Plasmid pFN33_tau and pFC36_tau gave the best signal to noise ratio of all combinations tested and are orientated antiparallel with regard luciferase large bit (17.6 kDa) and small bit (11 amino acids) fusions. Tau-Fusion protein expression is governed by the relatively weak HSV-thymidine kinase promoter, and has been verified by Western blotting (data not shown). After 16 h overnight incubation in serum-containing DMEM growth medium, medium was replaced with 30 µL Opti-MEM^TM^ comprising 1 µM, 10 µM inhibitors or 0.1% DMSO as vehicle control. Inhibitor treatment for 60 min at 37 °C was followed by addition of 5 µL luciferase substrate furimazine (2-furanylmethyl-deoxy-coelenterazine #N1130, 1:400 (*v*:*v*) diluted in Optimem, Promega). The luminescence signal has been measured immediately using Mithras LB 940 Multimode Microplate Reader (Berthold Technologies, Bad Wildbad, Germany). Background luciferase activity from nontransfected cells was subtracted and luminescence was normalized to vehicle control (0.1% DMSO). A 384-well plate was run in parallel were HuH-7 cells were transfected with pNL1.1.TK[Nluc/TK] vector expressing a constitutive active luciferase as a reference and inhibitor treatment was done accordingly. Luciferase readings from the tau interaction assay were normalized to the constitutive luciferase readings to remove unspecific effects [39]. All inhibitor treatments were repeated 8-fold.

## 4. Conclusions

The presently used drugs in AD therapy show a temporary benefit only in the early stage of the disease. A causative cure of AD is not possible with those drugs, as they allow only a symptomatic treatment. A novel approach for a causative therapy may be a reduction of the formation of harmful tau aggregation intermediates and tau filaments. A hyperphosphorylation of the tau protein that is discussed to contribute to the tau aggregate formation may be prevented by the use of protein kinase inhibitors. Within our first compound series, we identified nanomolar inhibitors of three kinases with relevance to tau pathology. Structural features which favour such activities are 3-hydroxy substitutions and an annelated phenyl residue attached to the benzofuropyridine scaffold. A reduction of tau protein phosphorylation could be demonstrated depending from the respective kinase activity of three relevant compounds with different activities towards the respective kinases. We could demonstrate a direct correlation between the structure-dependent compound ability to inhibit the relevant protein kinases and a reduced tau interaction in a luminescence based protein complementation assay.

Thus, the identified lead compounds may be a perspective tool for further preclinical tests to reduce tau aggregation based on a combination of diminished tau interaction and reduced tau phosphorylation.

## Figures and Tables

**Table 1:** Protein kinase affinities of target compounds **3a**–**e** and **4a**–**c**.

Cpd.	R	Protein Kinase Affinities (µM) ^a^		
		cdk1	cdk5/p25	gsk-3β
**3a**	**OEt**	0.17 ± 0.031	0.46 ± 0.022	0.083 ± 0.034
**3b**	**OBn**	2.30 ± 0.33	n.a. ^b^	5.80 ± 1.20
**3c**	**OMe**	0.14 ± 0.023	0.51 ± 0.012	0.062 ± 0.023
**3d**	**F**	1.40 ± 0.24	n.a. ^b^	0.38 ± 0.93
**3e**	**OH**	0.013 ± 0.0031	0.11 ± 0.043	0.024 ± 0.0021
**4a**	**OBn**	0.091 ± 0.012	2.10 ± 0.24	1.60 ± 0.23
**4b**	**F**	1.06 ± 0.63	160 ± 34	190 ± 37
**4c**	**OH**	0.77 ± 0.062	0.073 ± 0.012	0.012 ± 0.013

^a^ Mean of three determinations. ^b^ not active.

## Supplementary Materials

The following are available online, Figure S1: Predicted binding mode of compound **3e** (colored orange) at gsk-3β, Figure S2: Predicted binding mode of compound **4a** (colored magenta) at cdk1 and cdk5.
