# Supplementary material for: Novel Protein Kinase Inhibitors Related to Tau Pathology Modulate Tau Protein-Self Interaction Using a Luciferase Complementation Assay
Source: Molecules. 2018 Sep 12;23(9):2335. doi: 10.3390/molecules23092335 (PMC6225193; doi:10.3390/molecules23092335)
Supplement: Supplementary file 1 [file molecules-23-02335-s001.pdf]

Supplementary Information

Molecular docking

The inhibitor structures were built and energy minimized using MOE2010.10 (Chemical Computing Group, Inc.: Montreal, Canada, 2010) and the MMFF94 force field. The X-ray structures of cdk5 (PDB code 1UNL) and of gsk3β (PDB code 3GB2) were taken from the Protein Databank and the homology model of cdk1 was built using the closely related cdk2 X-ray structure in complex with a diaminopyrimidine inhibitor (PDB code 2FVD). Hydrogen atoms were added and the protein structure was minimized using the MMFF94 force field and position restraints on backbone atoms (force constant of 100 kcal/mol). Docking was carried out using the software GOLD 5.0 (Cambridge Crystallographic Data Centre, Cambridge, UK, 2011). Cys83(cdk5)/Leu83(cdk1)/Val135(gk3β) (located at the hinge region) were defined as center of the binding site with a radius of 15 Å. Water molecules located at the binding pocket were considered for docking using the 'toggle' mode within GOLD. Goldscore was used as scoring function to rank all docking poses.

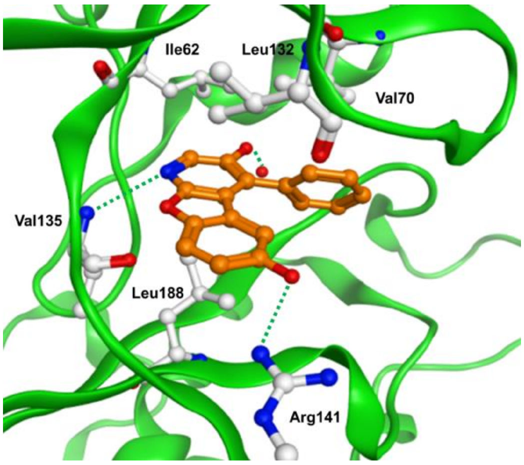

Figure S1. Predicted binding mode of compound **3e** (colored orange) at gsk-3β. The protein backbone is shown as ribbon and interacting amino acid residues are shown in cyan. Hydrogen bonds are displayed as dashed lines.

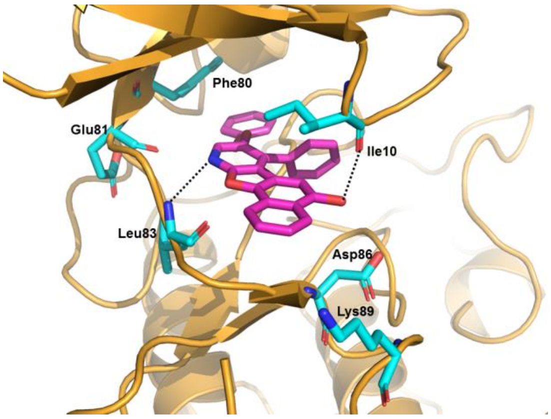

Figure S2a. Predicted binding mode of compound **4a** (colored magenta) at cdk1. The protein backbone is shown as ribbon and interacting amino acid residues are shown in cyan. Hydrogen bonds are displayed as dashed lines.

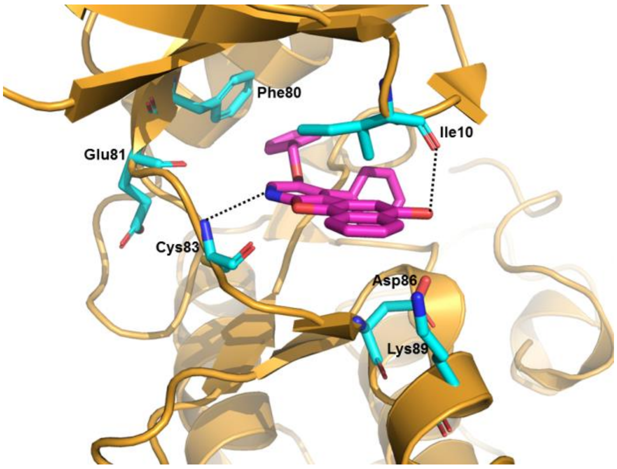

Figure S2b. Predicted binding mode of compound **4a** (colored magenta) at cdk5. The protein backbone is shown as ribbon and interacting amino acid residues are shown in cyan. Hydrogen bonds are displayed as dashed lines.
